# Biologically Equivalent Dose Comparison Between Magnetic Resonance-Guided Adaptive and Computed Tomography-Guided Internal Target Volume-Based Stereotactic Body Radiotherapy for Liver Tumors

**DOI:** 10.7759/cureus.33478

**Published:** 2023-01-07

**Authors:** Kiyoshi Yoda, Aya Sato, Yuta Miyake, Takeru Arato, William Starbuck

**Affiliations:** 1 Research Physics, Elekta K.K., Tokyo, JPN; 2 Application Training, Elekta K.K., Tokyo, JPN; 3 Application Physics, Elekta K.K., Tokyo, JPN; 4 Clinical Marketing, Elekta Pty Ltd., North Sydney, AUS

**Keywords:** virtual planning, isotoxic planning, liver tumor, biologically equivalent dose, mr-guided stereotactic body radiotherapy

## Abstract

Background and aim

Magnetic resonance (MR) imaging has been increasingly adopted in the field of radiotherapy, and the most advanced MR image-guided radiotherapy is known as MR-guided online adaptive radiotherapy (MRgOART), which integrates MRI and linac systems. Few attempts have yet been made to directly compare treatment outcomes between the MRgOART and standard computed tomography (CT)-guided radiotherapy (CTgRT). Besides, it is reported that the biologically equivalent dose (BED) may be a good predictor of the local control (LC) and the overall survival (OS) for liver tumors. The purpose of this study is to compare the BEDs between the MRgOART and the CTgRT by way of virtual isotoxic planning for liver tumors. The hypothesis of this study is therefore that the MRgOART increases LC and OS as compared to the CTgRT.

Materials and methods

Using the five patient cases available, isotoxic planning was performed. For CTgRT, an internal target volume (ITV) was defined, and the planning target volume (PTV) was created by adding an isotropic margin of 10 mm. For MRgRT, a gross tumor volume (GTV) was defined, and the PTV was created by adding an isotropic margin of 5 mm. Each tumor size was virtually adjusted so that the CTgRT plans resulted in BED <100 Gy under the condition that the nearest organs at risk receive maximum tolerated doses. Subsequently, the BED was recalculated for MRgOART plans with the adjusted tumor size.

Results and discussion

It was found that the BEDs of the MRgOART plans always exceeded 100 Gy and were approximately 20 Gy larger than those of the corresponding CTgRT plans. Literature shows that superior overall survival rates for liver tumors were observed when BED was >100 Gy as compared to BED <100 Gy, suggesting that MR-guided adaptive planning may potentially lead to better treatment outcomes for liver tumors. We have also observed a case where the duodenum largely moved and abutted the liver after the CT images were acquired, indicating a significant disadvantage of the standard CTgRT because such abutting is not observable by the cone-beam CT immediately before treatment.

Conclusion

A highly accelerated evidence-creation procedure to suggest the clinical superiority of MRgOART has been arguably proposed with promising results. The sample size is small and limits the extent to which the findings in this study can be generalized. Further virtual clinical trials within the radiotherapy community are awaited with more clinical outcomes data.

## Introduction

Magnetic resonance (MR) imaging has been increasingly adopted in the field of radiation treatment, and the most advanced MR image-guided radiotherapy is known as MR-guided online adaptive radiotherapy (MRgOART), which integrates MRI and linac systems [1-5]. However, few attempts have yet been made to directly compare treatment outcomes such as local control and overall survival between the MRgOART and the standard linac-based stereotactic body radiotherapy (SBRT), besides the MR-guided adaptive versus ITV-based stereotactic body radiotherapy for hepatic metastases (MAESTRO) randomized controlled trial for the liver metastases [6].

It was reported that the local control (LC) and the overall survival (OS) were associated with the biologically equivalent dose (BED) to tumors, for example, non-small cell lung cancer [7] and hepatocellular carcinoma [8-10], where the LC and the OS of these tumors were significantly higher when the BED was >100 Gy as compared to the BED <100 Gy. Considering these findings in the publications, the present authors endeavor to propose a highly accelerated evidence generation procedure where the BEDs are calculated and compared between the gross tumor volume (GTV)-based MRgOART and internal target volume (ITV)-based CT guided radiotherapy (CTgRT) by referring to five sets of liver tumor cases as an initial study. In each patient case, the tumor size is adjusted so that the BED on the CTgRT remains below 100 Gy to simulate less favorable cases from the real-world patient population. The hypothesis of this virtual planning study is that the MRgOART increases the LC and the OS for the above unfavorable cases with the CTgRT.

## Materials and methods

Five sets of anonymized liver tumor data were used in this study, with each set having a simulation CT scan acquired a few days before MRgOART and five MR data acquisitions on five treatment days, consistently under abdominal compression. All of the structures, including ITVs and GTVs, were already contoured in the CT and MR data. Using these data, ITV-based CTgRT and GTV-based MRgOART planning were performed with a prescribed dose of 50 Gy in five fractions (prescribed BED10 of 100 Gy, $\alpha /\beta$ = 10 Gy) and the dose constraints to the OARs employed previously [6]. The BED10 was calculated by a formula given in the appendices of this paper. Only in cases four and five were the normal liver dose constraints replaced with those recommended in the UK consensus report [11].

For the CTgRT, a planning target volume (PTV) was defined by adding an isotropic margin of 10 mm to the ITV [6]. For the MRgOART, PTVs were adaptively defined by adding an isotropic margin of 5 mm to the GTVs on the five-fraction data, assuming either gating or breath-holding [12]. Monaco treatment planning system 5.51 (Elekta, Stockholm, Sweden) having beam models of a VersaHD linac (Elekta, Stockholm, Sweden) and a Unity MR-linac (Elekta, Stockholm, Sweden) was employed for the treatment planning. A single-arc volumetric modulated arc therapy (VMAT) was employed for the CTgRT, whereas nine-beam intensity-modulated radiotherapy (IMRT) was used for the MRgOART.

To test our hypothesis, the following steps were taken: 1) A virtual CT-PTV volume was iteratively expanded and used for planning until a CT-PTV volume received the BED10 <100 Gy to simulate a less favorable case while maintaining all the organs at risk (OARs) constraints previously employed [6,11]. In reality, this manipulation may correspond to a growing tumor due to a late diagnosis. 2) This final CT-PTV volume was used to derive a final MR-PTV, where the final MR-PTV had an isotropic margin that was 5 mm less than the final CT-PTV.

To calculate dose distributions on MR images, a bulk electron density approach was used by referring to each patient’s organ-averaged electron densities calculated from corresponding CT images. The PTV D95 (minimum dose delivered to 95% of the PTV) was obtained for the BED calculation. In the MRgOART isotoxic planning, the D95s varied among five fractions and therefore mean D95 was used for the BED calculation. Finally, BED10s in the five cases were compared between the CTgRT and the MRgOART.

## Results

**Figure 1:**
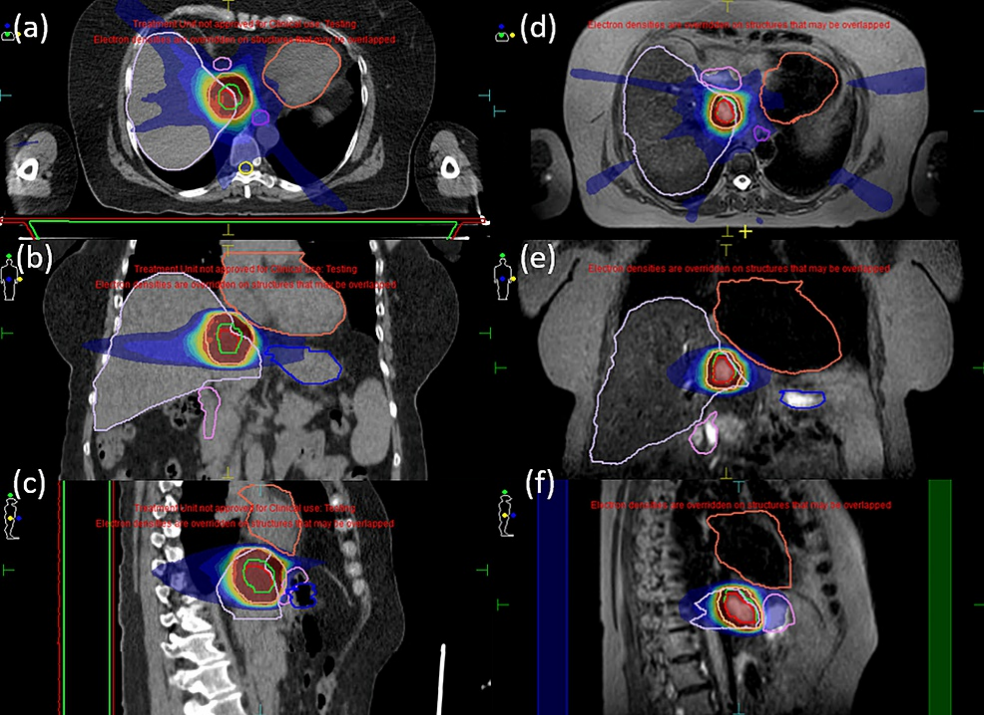
CTgRT and MRgOART isotoxic planning comparisons for case one. (a)–(c): CTgRT plan; (d)–(f): MRgOART plan on the first treatment day. The colors are liver in purple, heart in vermilion, duodenum in pink, stomach in blue, PTV in orange, ITV in yellow-green, and GTV in red. To simulate an unfavorable case (BED10 <100 Gy), the CT-PTV was expanded by 1 mm.

Figures 1a-1c show a calculated planning result for case one on three orthogonal CT planes for the CTgRT. The original CT-PTV resulted in a BED10 of 100 Gy, and therefore the CT-PTV was expanded by 1 mm to obtain a BED10 <100 Gy. Figures 1d-1f depict the planning result for case one (on the first day) on nearly identical three orthogonal MR planes.

**Figure 2:**
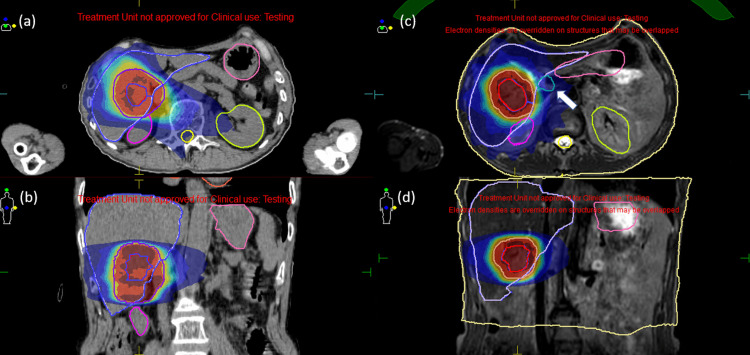
Isotoxic planning comparisons between CTgRT and MRgOART for case two. (a)-(b): CTgRT plan; (c)-(d): MRgOART plan (the first fraction). The duodenum was not adjacent to the liver in the CT image (invisible on these planes), whereas the duodenum in cobalt blue (white arrow) moved and abutted the liver at the time of MR image acquisition on the first treatment day.

Figures 2a-2b illustrate a CTgRT plan for case two while Figures 2c-2d indicate an MRgOART plan for the same case. The duodenum (in cobalt blue) was not adjacent to the liver in the CT image (invisible on these planes), whereas the duodenum moved and abutted the liver in the MR image (white arrow) on the first treatment day. The CT-PTV required an expansion of 4 mm for BED10 <100 Gy because no OARs were close to the target.

**Figure 3:**
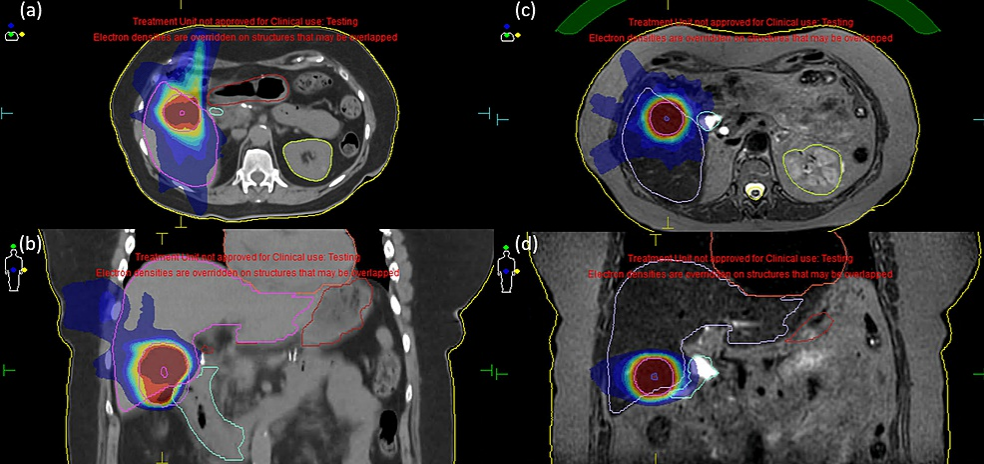
Isotoxic planning comparisons between CTgRT and MRgOART for case three. The tumor was very small, and a large CT-PTV expansion of 10 mm was required to obtain a BED10 <100 Gy.

Figures 3a-3b show a CTgRT plan, and Figures 3c-3d indicate an MRgOART plan for case three, where the tumor was very small and a large CT-PTV expansion of 10 mm was required for BED10 <100 Gy.

**Figure 4:**
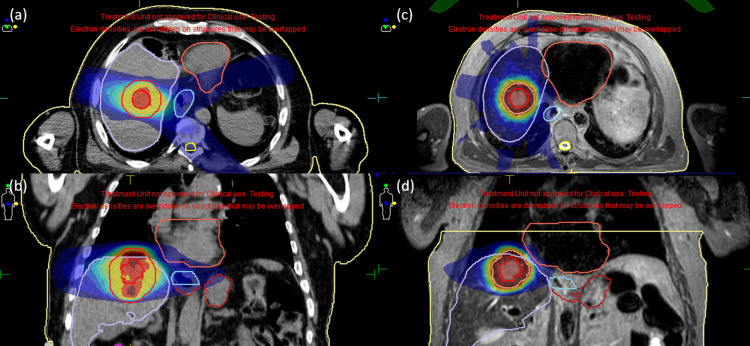
Isotoxic planning comparisons between CTgRT and MRgOART for case four. The tumor was located sufficiently far from the OARs. In this case, the normal liver was considered the most influential OAR.

Figures 4a-4b illustrate a CTgRT plan, and Figures 4c-4d demonstrate an MRgOART plan for case four, where the tumor was located far from OARs. In this case, the normal liver was considered the most influential OAR. It was therefore decided to employ the mean dose constraint for the normal liver that was recommended in the UK consensus report as an optimal constraint for five-fraction SBRT [11]. The tumor location in case five was similar to that in case four and is thus not shown here for brevity.

Table 1 shows the original GTV and ITV volumes with the CT-PTV expansion for BED10 <100 Gy. The calculated constrained doses to the most critical organs are also shown.

**Table 1:** Original GTV and ITV volumes, virtually added PTV expansion, declared dose constraint, and calculated constrained dose in each case. GTV: gross tumor volume; ITV: internal target volume; PTV: planning target volume; D0.5cc: the minimal dose received by the highest irradiated volume of 0.5 cc; Dmean: mean dose

	Case one	Case two	Case three	Case four	Case five
GTV (cc)	7.7	14.6	0.21	10.0	3.1
ITV (cc)	11.9	27.2	0.77	18.3	8.0
Virtually added PTV expansion	1 mm	4 mm	10 mm	3 mm	10 mm
Declared dose constraint	Duodenum	Duodenum	Duodenum	Normal liver	Normal liver
	D0.5cc <35 Gy	D0.5cc <35 Gy	D0.5cc <35 Gy	Dmean <13 Gy	Dmean <13 Gy
Calculated constrained dose	34.1 Gy	34.4Gy	34.1 Gy	12.6 Gy	12.1 Gy

Table 2 indicates the calculated D95 (Gy) of the CTgRT and MRgOART five-fraction plans for cases one to five. In case two, the distances between the tumor and the duodenum in the MR images were much smaller than those in the CT images, thereby reducing the D95s in the MR plans compared to other cases. In case three, the MR data for one fraction were not available.

**Table 2:** Calculated PTV D95 (Gy) in the CTgRT and MRgOART plans for cases one to five. In case three, the MR data for one fraction were not available. MR1–5: MR images acquired in the first to fifth fractions

	Case one	Case two	Case three	Case four	Case five
CT	46.33	46.57	45.56	35.93	44.31
MR1	52.83	50.34	52.34	52.60	53.04
MR2	54.89	50.20	52.43	52.18	52.80
MR3	51.53	50.35	52.25	52.65	52.85
MR4	53.78	50.13	52.12	52.26	53.11
MR5	53.35	50.40		52.64	53.20

Table 3 shows BED comparisons in the five liver cases between CTgRT and MRgOART under isotoxic planning conditions. The BEDs were calculated assuming $\alpha /\beta$ = 10 Gy. The difference in the median BED10s among the five cases between the two arms was 20.4 Gy, whereas the median of the BED10 differences (denoted as $\Delta$BED10) between the two arms among the five cases was 20.7 Gy.

**Table 3:** BED comparisons between CTgRT and MRgOART in the five liver cases under isotoxic planning conditions. CTgRT: CT-guided radiotherapy; MRgOART: MR-guided online adaptive radiotherapy; $\Delta$BED10: the difference in biologically effective doses ($\alpha /\beta$ = 10 Gy) between CTgRT and MRgOART

	Case one	Case two	Case three	Case four	Case five	Median
CTgRT	89.3 Gy	89.9 Gy	87.1 Gy	61.8 Gy	83.6 Gy	87.1 Gy
MRgOART	110.0 Gy	100.9 Gy	107.0 Gy	107.5 Gy	109.2 Gy	107.5 Gy
$\Delta$BED10	20.7 Gy	11.0 Gy	19.9 Gy	45.7 Gy	25.6 Gy	20.7 Gy

## Discussion

In this virtual planning study, the tumor size was adjusted or expanded to create a typical unfavorable case with a BED10 <100 Gy using standard CT-guided radiotherapy under the condition that the most critical OAR receives the upper limit of the dose tolerance. In other words, we cannot achieve BED10 >100 Gy in these cases because the dose cannot be escalated. If the tumor is larger than the tested cases, the BED will be further reduced under identical dose constraints. Conversely, if the tumor is smaller, the dose constraints may be met with BED10 >100 Gy for CTgRT. We only tested five cases; however, it is conceived that the virtual planning comparison between different treatment modalities may be highly efficient to acquire knowledge about the advantages of the MRgOART as compared to the standard CTgRT by way of the tumor size adjustments.

If we look at the field of radiology, a terminology called "virtual clinical trial" exists in that computer simulation studies are performed using precise digital human phantoms and accurate Monte Carlo calculations [13]. This may work well for diagnostic hypothesis validation. In the field of radiotherapy, the endpoints are mostly treatment outcomes, which currently have no certified digital models. This means that we need retrospective data that may connect simulation and outcomes.

Literature shows that superior LC and OS for liver tumors were observed when BED10 was >100 Gy as compared to BED10 <100 Gy [8-10]. In these reports, the median BED dose differences between the two arms were approximately 20 Gy. In our study, it was found that the median BED dose differences between the CTgRT and MRgOART among the five cases were also approximately 20 Gy, suggesting that MR-guided adaptive planning may potentially lead to better treatment outcomes for liver tumors if we refer to the previous findings.

Recently, an LC model for metastatic liver tumors was proposed based on a tumor control probability formula, showing that the two-year LC after SBRT had increased monotonically as a function of the BED up to 300 Gy [14]. This may suggest that MRgOART for metastatic liver tumors would be superior in these cases even when CTgRT would achieve a BED10 >100 Gy, as MRgOART would provide higher BEDs due to smaller PTV margins under isotoxic conditions.

In case two, it was observed that the duodenum moved between the CT acquisition day and the first treatment day. This type of movement of the duodenum is occasionally observed, clearly demonstrating a significant disadvantage of the standard CTgRT for abdominal SBRT because it is not detectable using cone-beam CT acquired immediately before the beam delivery.

In case four, the D95 of the CTgRT was significantly reduced after employing a normal liver dose constraint of Dmean (mean dose) <13 Gy in place of D700cc (the minimum dose in the most irradiated 700 cc volume) <24 Gy, indicating that the former constraint recommended in the UK consensus report [11] is more strict.

The BED for the MRgOART was calculated based on the mean D95 among the five fractions. An exact formula has been derived and shown in the appendices. It was found that the difference between the approximation using the mean D95 and the exact calculation was negligibly small except for much larger D95 fluctuations and much smaller $\alpha /\beta$ such as 1 or 2 Gy, the differences being enhanced by the second order term of the fraction doses in the equation (2). An obvious limitation of this study is the number of cases considered. A larger number of clinical cases may be required for a more precise simulation of the real-world patient population.

## Conclusions

A highly accelerated evidence-creation procedure to suggest the clinical superiority of MRgOART has been arguably proposed with promising results. The sample size is small and limits the extent to which the findings in this study can be generalized. We have also observed a case where the duodenum largely moved and abutted the liver after CT image acquisition, indicating a significant disadvantage of standard CT-guided radiotherapy because such abutting is not observable by cone-beam CT imaging at the time of treatment. Further virtual clinical trials within the radiotherapy community are awaited with more clinical outcome data.

## Appendices

An exact BED formula for isotoxic planning:

When fraction doses during a course of radiotherapy are given as di where i denotes fraction number ranging from 1 to n, the total survival fraction S of the tumor cells is given below:

(1)

$$S=\prod_{i=1}^n e^{-\alpha d_{i}-\beta d_i^2}=e^{-\alpha (\sum_{i=1}^n d_{i}+\frac{\beta}{\alpha}\sum_{i=1}^nd_i^2)}$$

By definition [15], BED is given as follows where the summation goes from i=1 to n.

(2)

$$BED=\sum_{i=1}^nd_{i}+\frac{\beta}{\alpha}\sum_{i=1}^nd_i^2$$

It is noted that equation (2) goes to the usual BED formula [15] when the fraction doses are identical.
